# An Inducible Neural Stem Progenitor Cell Model for Testing Therapeutic Interventions Against Neurodegeneration FENIB

**DOI:** 10.1002/ddr.70041

**Published:** 2025-01-03

**Authors:** Alessandro Giustini, Alice Maiocchi, Ilaria Serangeli, Martina Pedrini, Anna Quintiliani, Valentina Sabato, Francesca Bonato, Pierfausto Seneci, Giuseppe Lupo, Daniele Passarella, Elena Miranda

**Affiliations:** ^1^ Department of Biology and Biotechnologies ‘Charles Darwin’ Sapienza University of Rome Rome Italy; ^2^ Department of Chemistry University of Milan Milan Italy; ^3^ Present address: Veneto Institute of Molecular Medicine Padova Italy

**Keywords:** cereblon, embelin, FENIB, neural progenitor cells, neuroserpin, PROTAC

## Abstract

Familial encephalopathy with neuroserpin inclusion bodies (FENIB) is a neurodegenerative pathology caused by accumulation of mutant neuroserpin (NS) polymers inside the endoplasmic reticulum (ER) of neurons, leading to cellular toxicity and neuronal death. To date, there is no cure for FENIB, and only palliative care is available for FENIB patients, underlining the urgency to develop therapeutic strategies. The purpose of this work was to create a cellular system designed for testing small molecules able to reduce the formation of NS polymers. Our results show the generation and characterisation of a novel cell culture model for FENIB based on neural stem progenitor cells (NPCs) with inducible expression of either wild type (WT) or G392E NS, a variant that causes severe FENIB. We also report the use of these novel cell lines to explore the effects of four different proteolysis targeting chimaera (PROTAC) compounds, small bivalent molecules engineered to bind to the E3 ubiquitin ligase cereblon, and to NS through a recruiting motif based on the small molecule embelin. This approach aims to enhance the degradation of mutant NS after retro‐translocation to the cytosol by facilitating its targeting to the proteasome. Our results show little toxicity and no variation in NS levels with any of the compounds tested. In conclusion, this work sets the basis for future attempts to identify molecules able to prevent NS accumulation inside the ER of cultured cells.

**Abbreviations**
ACN: acetonitrile
CAN: ceric ammonium nitrate
Cs_2_CO_3_
: cesium carbonate
DCM: dichloromethane
DIPEA: *N*,*N*‐diisopropylethilamine
DMF: *N*,*N*‐dimethylformamide
ER: endoplasmic reticulum
ERAD: ER associated degradation
EtOAc: ethyl acetate
H_2_O_2_
: hydrogen peroxide
H_2_SO_4_
: sulfuric acid
HATU: hexafluorophosphate azabenzotriazole tetramethyl uronium
K_2_CO_3_
: potassium carbonate
MeI: iodomethane
MeOH: methanol
Na_2_SO_4_
: sodium sulphate
NS: neuroserpin
*n*‐BuLi: *n*‐butyllithium
*n*‐Hex: *n*‐hexane
PAGE: polyacrylamide gel electrophoresis
rt: room temperature
TFA: trifluoroacetic acid
THF: tetrahydrofuran
WT: wild type

## 1 Introduction

The autosomal dominant dementia familial encephalopathy with neuroserpin inclusion bodies (FENIB) (Davis et al. 1999) is a neurodegenerative pathology caused by intracellular accumulation of mutant variants of neuroserpin (NS), an extracellular inhibitor of the serine protease tissue plasminogen activator that is mainly produced by neurons and secretory cells of neural origin (D'Acunto et al. 2021). NS is a member of the serpin superfamily, a large and conserved group of proteins that control the activity of serine proteases. Their suicide inhibitory mechanism is based on the flexibility of the metastable serpin molecule, which renders serpin proteins vulnerable to mutations that increase their natural instability, altering their folding, and leading to the formation of polymeric chains within the endoplasmic reticulum (ER), where these polymers can aggregate to form inclusion bodies. The mechanism of NS polymer formation is still unknown, but may be similar to that of the archetypal serpin alpha‐1 antitrypsin, based on repetitive swapping of the C‐terminal domain of consecutive molecules in the polymeric chain (Faull et al. 2020; Yamasaki et al. 2011). The hallmark of FENIB is the presence of intraneuronal inclusion bodies composed mostly of NS polymers across the central nervous system, mainly in the cerebral cortex, substantia nigra and hippocampus (Coutelier et al. 2008; Davis et al. 2002, 1999). The first four mutations identified in FENIB patients display a strong genotype‐phenotype correlation (from less to more severe: Ser49Pro, Ser52Arg, His338Arg and Gly392Glu), driving NS polymerisation at a rate directly proportional to the number of inclusions found in the brain, and inversely correlated to the age of onset of disease (Davis et al. 2002; Miranda, Römisch, and Lomas 2004, 2008). NS polymer accumulation causes toxicity in a fly model of FENIB (Miranda et al. 2008), but how this happens at the molecular level remains poorly understood. Recently, expression of the severe G392E variant of NS in cultured neurons differentiated from mouse neural progenitor cells (NPCs) has shown that NS polymers induce oxidative stress and mitochondrial alterations as part of the toxic insult (D'Acunto et al. 2021; Guadagno et al. 2017).

Being a secretory protein, NS is co‐translationally inserted in the ER, where it folds to acquire its native conformation. In the presence of mutations that alter its correct folding, part of the monomeric misfolded protein is recognised by the ER quality control system and gets degraded by ER associated degradation (ERAD) (Kroeger et al. 2009; Miranda, Römisch, and Lomas 2004), following the pathway for luminal ER proteins that involves gp78 and Hrd1 (Ying et al. 2011). Monomeric mutant NS that escapes ERAD are partly secreted, while the rest undergoes polymerisation, linking into chains that are highly ordered and stable, and accumulate within the ER forming inclusion bodies. An approach that accelerates proteasomal degradation of monomeric mutant NS may be of therapeutic value for FENIB patients. PROTACs (PROteolysis TArgeting Chimaeras) are heterobifunctional compounds that comprise two small molecules, one able to bind the target protein (protein of interest, POI) and one able to recruit an E3 ubiquitin ligase, joined by a linker. By temporarily neighbouring the target protein and the E3 ligase, proteasomal degradation of the target protein should be greatly accelerated. E3 ligases recruited by current PROTACs, including cereblon (Ito 2024) and von Hippel‐Lindau (Wang et al. 2022), have a broad ubiquitination capacity, and have been successfully applied to cytosolic target proteins involved mainly in cancer, such as oestrogen and androgen receptors (Békés, Langley, and Crews 2022; Sakamoto et al. 2003).

In this work we report the production and characterisation of a novel cellular model of FENIB based on constitutive, inducible expression of either wild type (WT) or G392E NS in mouse NPCs, under the control of a doxycycline responsive promoter. We also report a first attempt to apply this system to the testing of PROTAC molecules specifically designed to promote the proteasomal degradation of NS.

## 2 Materials and Methods

### 2.1 Generation and Characterisation of a Novel Inducible Neural Model of FENIB

#### 2.1.1 Generation of ePiggyBac Plasmids for Expression of NS

Two plasmids were produced by cloning either WT or G392E NS in the integrative, inducible ePiggyBac (ePB) plasmid (Rosa et al. 2014), which contains a reverse tetracycline‐controlled transactivator and TET‐responsive elements under a constitutive promoter, as well as a puromycin resistance gene for selection of mammalian cells. Previously made pTP6 plasmids expressing human WT and G392E NS under a constitutive promoter (Guadagno et al. 2017) were amplified in XL‐1 blue competent cells (Agilent, 200130) and plated in LB (Luria‐Bertani) agar under ampicillin selection at 1 μg/mL. Transformed colonies were picked and amplified in LB broth and plasmid DNA was purified with the QIAGEN Plasmid Midi Kit following the manufacturer's protocol (QIAGEN, 12123). Purified pTP6 and ePB plasmids were digested with BamH1 and NotI restriction enzymes for 10 min at 37°C, run on a 1% agarose gel, and the ePB and NS bands cut and purified with the NucleoSpin Gel and PCR Clean‐up XS kit (Macherey‐Nagel, and 740611.50). Purified DNAs were ligated to insert the NS sequences under the control of TET responsive elements of the ePB plasmid, transformed into XL1‐blue competent cells, and purified. Correct ligation was ascertained by Bam/Not restriction and analysis on agarose gel.

#### 2.1.2 Generation of NPC Lines With Inducible Expression of NS

Early passage NPCs previously obtained from embryonic cortex of E13.5 mice (Soldati et al. 2012) were transfected with ePB‐Puro‐TT‐NS plasmids (ePB, Lenzi et al. 2016) with an Amaxa Nucleofector unit using the Neural Stem Cell Nucleofector kit (Lonza, VPG‐1004). One million cortical early passage mouse NPCs were transfected with 2 µg of ePB‐WT NS or ePB‐G392E NS plasmid DNA and a plasmid encoding a transposase in a 1:10 ratio. Two days after transfection, NPCs with stable integration of the ePB‐NS transgenes were selected with 1 μg/mL puromycin (Merck, Sigma‐Aldrich, P8833).

#### 2.1.3 NPC Culture and Treatments

NPCs were maintained under proliferative conditions seeding them in T25 flasks (Corning, 430639) coated with 10 μg/mL poly‐ornithine (Merck, Sigma‐Aldrich, P3566) and 5 μg/mL laminin (Merck, Sigma‐Aldrich, 354232), using basal medium [DMEM/F12 (Thermo Fisher Scientific, 32500035), 0.892 M NaHCO_3_ (Merck, Sigma‐Aldrich, S8761), 1 mM Hepes (Merck, Sigma‐Aldrich, H0887), 0.1 M GlutaMAX (Thermo Fisher Scientific, Gibco, 35050038), 0.033 M glucose (Merck, Sigma‐Aldrich, G8769), 0.1 M penicillin/streptomycin (Thermo Fisher Scientific, Gibco, 15140122)] supplemented with 20 ng/mL human epidermal growth factor (hEGF, R&D, 236‐EG‐01M), 10 ng/mL basic fibroblast growth factor (bFGF, R&D, 4114‐TC‐01M), B‐27 2% v/v (Thermo Fisher Scientific, 17502048) and 1% v/v N2 (Thermo Fisher Scientific, 17502048) to make complete medium. For the induction of NS expression, doxycycline (Merck, Sigma‐Aldrich, D5207) was added at 500 ng/mL. Upon confluence, cells were dissociated with Accutase (Corning, 25‐058‐CI) and re‐seeded on fresh flasks. Cells were kept at 37°C in a 5% CO_2_ atmosphere.

#### 2.1.4 Cellular Viability MTT Assay

Cells were seeded at 100,000 cells per well of a coated 24 well plate in complete medium as described above. The following day, NS expression was induced by replacing the medium with fresh complete medium containing 500 ng/mL of doxycycline. 1 h after NS induction, putative PROTAC compounds, previously dissolved in DMSO, were added to the wells, starting from an initial concentration of 20 µM and performing 1:2 dilutions directly in the culture wells. PROTAC compounds were incubated for 24 h, and cell viability was assessed using the MTT colorimetric assay following the manufacturer's protocol (Abcam, ab211091).

#### 2.1.5 Denaturing and Non‐Denaturing Polyacrylamide Gel Electrophoresis (PAGE) and Western Blot

Analysis of cell lysates and culture medium supernatants from NPC‐NS cells was carried out using denaturing and non‐denaturing PAGE and western blot protocols described before (Belorgey et al. 2011). Briefly, the culture media were collected by centrifugation at 500 g, RT for 10 min. The cells were lysed with 80 μL of lysis buffer (150 mM NaCl, 50 mM Tris‐Cl, pH 7.5, 1% v/v Nonidet P‐40, plus 1 mM protease inhibitor cocktail), and the soluble fraction was collected by centrifugation at 17.000 g, 4°C for 15 min. For denaturing PAGE, samples were mixed with loading buffer containing 10% v/v beta‐mercaptoethanol and 4% w/v SDS and heated at 95°C; for non‐denaturing PAGE, samples were mixed with loading buffer without denaturants. Samples were analysed by 10% w/v acrylamide SDS‐PAGE and non‐denaturing 7.5% w/v acrylamide PAGE, followed by electrotransfer (omitting the methanol in the transfer buffer), and western blot with purified rabbit anti‐NS polyclonal antibody made in house at 1 µg/mL (Miranda et al. 2008), mouse anti‐actin antibody (Millipore, MAB1501), anti‐rabbit‐HRP (Merck, Sigma‐Aldrich, AP106P) and anti‐mouse‐HRP secondary antibodies (Merck, Sigma‐Aldrich, A9044). The HRP signal was developed using LiteUP and TURBO extra sensitive chemiluminescent substrates (Euroclone S.p.A., EMP002005 and EMP012001 respectively), and visualised on a ChemiDoc system (BioRad).

#### 2.1.6 Immunofluorescence Staining and Analysis

Immunofluorescence analysis of NPC‐NS cells was carried out as described before (Belorgey et al. 2011). Briefly, cells were grown on coverslips treated with poly‐ornithine and laminin as described above, placed in 24‐well plates. Cells were washed with PBS (phosphate buffered saline, Corning, 21‐031‐CV), fixed in ice‐cold 4% (w/v) paraformaldehyde (Thermo Fisher Scientific, 043368.9 M) for 30 min at RT and washed again. Cells were then treated with permeabilization/blocking solution (0.5% Triton, 1% BSA, 0.1% sodium azide in PBS) for 1 h, and immunostained with a rabbit anti‐NS polyclonal antibody made in house (Miranda et al. 2008), anti‐KDEL (Enzo Life Sciences, ADI‐SPA‐827), and secondary antibodies goat anti‐rabbit IgG‐Alexa Fluor 594 (Abcam, 150080) and goat anti‐mouse IgG‐Alexa Fluor 488 (Abcam, 150113). Nuclear DNA was stained with DAPI (Merck, Sigma‐Aldrich, D9542). Coverslips were mounted with FluorSave (Calbiochem, VWR International, 345789‐20) plus 2% DABCO. Imaging was performed on a Zeiss Axio Observer microscope.

### 2.2 Synthesis of PROTAC Compounds

The materials and methods applied to the synthesis of PROTAC compounds are described in the supplementary methods section.

## 3 Results

### 3.1 Generation of an Inducible Neuronal Model of FENIB

The cell culture models of FENIB produced so far are based on constitutive expression of WT or polymerogenic mutant NS (Ingwersen et al. 2021; Miranda, Römisch, and Lomas 2008, 2004; Roussel et al. 2013), and thus are less suitable for analysis of NS degradation since NS is constantly produced and, in the case of the mutant FENIB variants, deposited as polymers within the ER, and this may mask the enhanced degradation elicited by PROTACs. We therefore created an inducible expression cell system based on mouse NPCs stably engineered for inducible expression of WT or G392E NS, under a TET‐ON system that allowed us to finely modulate NS expression. Early passage NPCs from E13.5 mouse embryos were electroporated with Piggy Bac (ePB) plasmids containing either WT or G392E NS, creating NPC WT NS and NPC G392E NS polyclonal cell lines.

**Figure 1:**
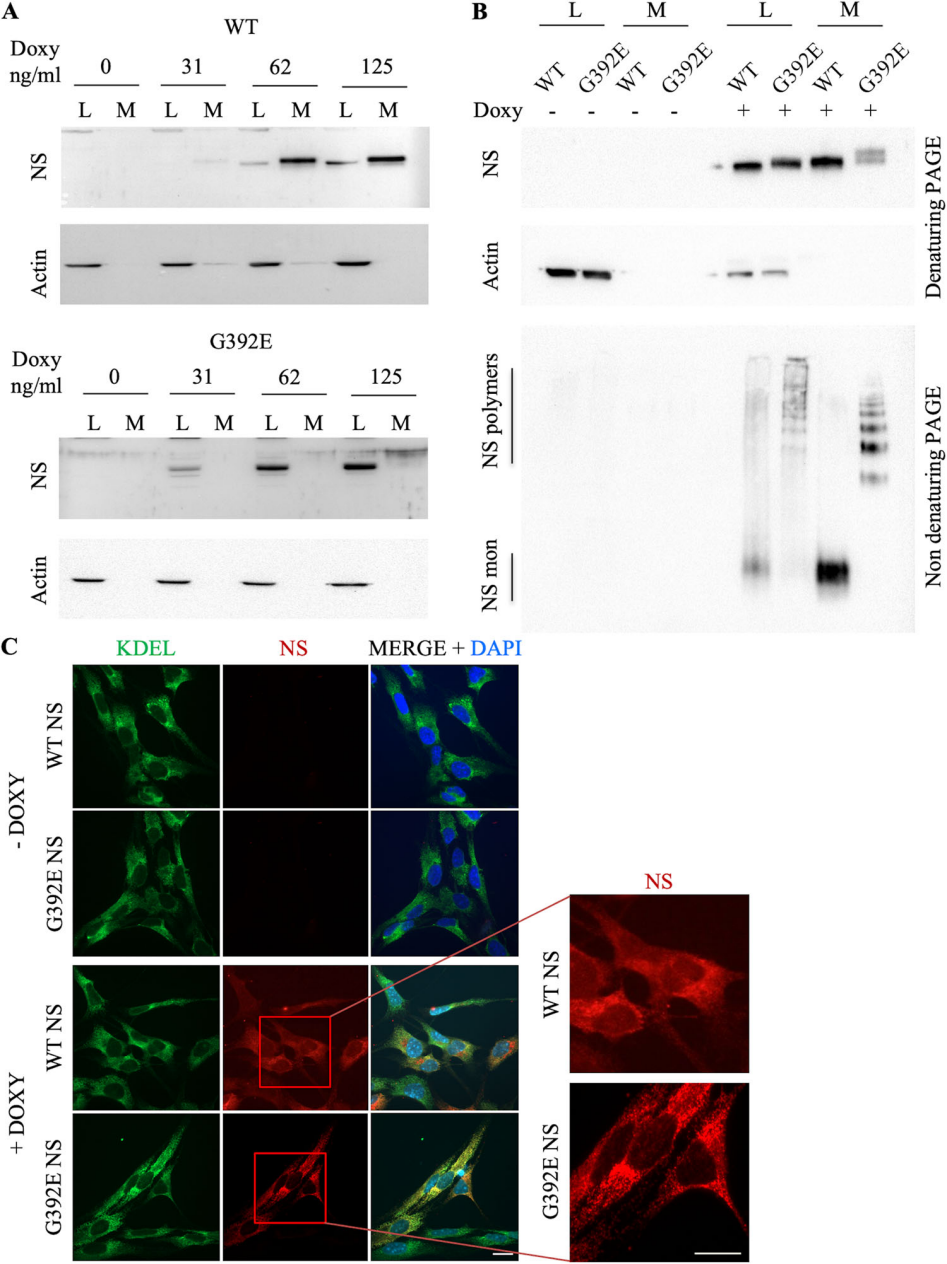
Characterisation of the novel inducible cellular model of FENIB. (A) Cell lysates (L) and culture medium supernatants (M) of NPCs expressing WT (upper panels) or G392E (lower panels) treated with increasing concentrations of doxycycline (Doxy) were analysed by SDS‐PAGE followed by western blot using an anti‐NS polyclonal antibody, or an anti‐actin antibody as a loading control. (B) Cell lysates and culture medium supernatants of NPCs expressing WT or G392E NS, induced or not with doxycycline 125 ng/mL for 24 h, were analysed by SDS‐ (top and middle panels) and non‐denaturing (bottom panel) PAGE and western blot, using anti‐NS polyclonal and anti‐actin (loading control) antibodies. (C) NPCs expressing either WT or G392E NS were grown on treated glass coverslips, fixed and stained with antibodies against the ER retention motif KDEL (green staining, left column panels) and NS (red staining, middle column panels). The right column panels show the overlap of both signals (merge), with the nuclear DNA stained with DAPI for reference. Scale bar: 10 µm. The enlarged panels show a detail of the diffuse staining for WT NS and the punctuated staining typical of polymerogenic variants for G392E NS.

To assess NS expression, we first performed SDS‐PAGE followed by western blot analysis of cell lysates and culture medium supernatants of NPC NS cells induced with increasing amounts of doxycycline for 24 h, as shown in Figure 1A. We observed a tight control of NS expression, with no signal in the absence of doxycycline, and selected 125 ng/mL as the best doxycycline concentration for induction. We next performed SDS‐ and non‐denaturing PAGE and western blot analysis of cell lysates and culture media of cells induced for 24 h. As shown in Figure 1B, NS expression was observed exclusively when cells were treated with doxycycline, even if the lysates of non‐induced cells showed a higher protein content as assessed with an anti‐actin loading control antibody (middle panel). With denaturing PAGE analysis (top panel), a strong signal for WT NS was observed both in cell lysates and culture medium, while G392E NS showed a strong signal in cell lysates but a weaker one in the culture medium, in agreement with the lower rate of secretion associated to intracellular polymerisation and accumulation of this mutant variant in the ER (Ingwersen et al. 2021; Miranda et al. 2008; Moriconi et al. 2015). G392E NS appeared as a double band in the culture medium, due to the presence of two or three N‐glycan chains as earlier described (Moriconi et al. 2015). When analysed by non‐denaturing PAGE (bottom panel, native PAGE), WT NS appeared as a monomer both in cell lysates and culture medium, while G392E NS was found as a ladder of polymers of increasing size both in the intracellular and extracellular fractions.

The correct expression and intracellular localisation of NS were assessed by immunofluorescence (Figure 1C). NS was not detected in non‐induced cells, while a strong NS signal was evident in cells treated with doxycycline (125 ng/mL for 24 h). WT NS was observed as a diffused red staining (enlarged panel) that partially co‐localised with the ER retention sequence KDEL; a discreet and intense NS signal nonoverlapping with the KDEL one was observed in many cells, in agreement with a strong presence of WT NS in the Golgi apparatus as already described (Miranda, Römisch, and Lomas 2008, 2004; Moriconi et al. 2015). G392E NS showed as a strong staining that extensively co‐localised with the ER marker, with a punctate distribution typically correlated with polymer accumulation within the ER (enlarged panel) (Miranda, Römisch, and Lomas 2008, 2004; Moriconi et al. 2015).

### 3.2 Synthesis of PROTAC Compounds That Target NS

**Figure 2:**
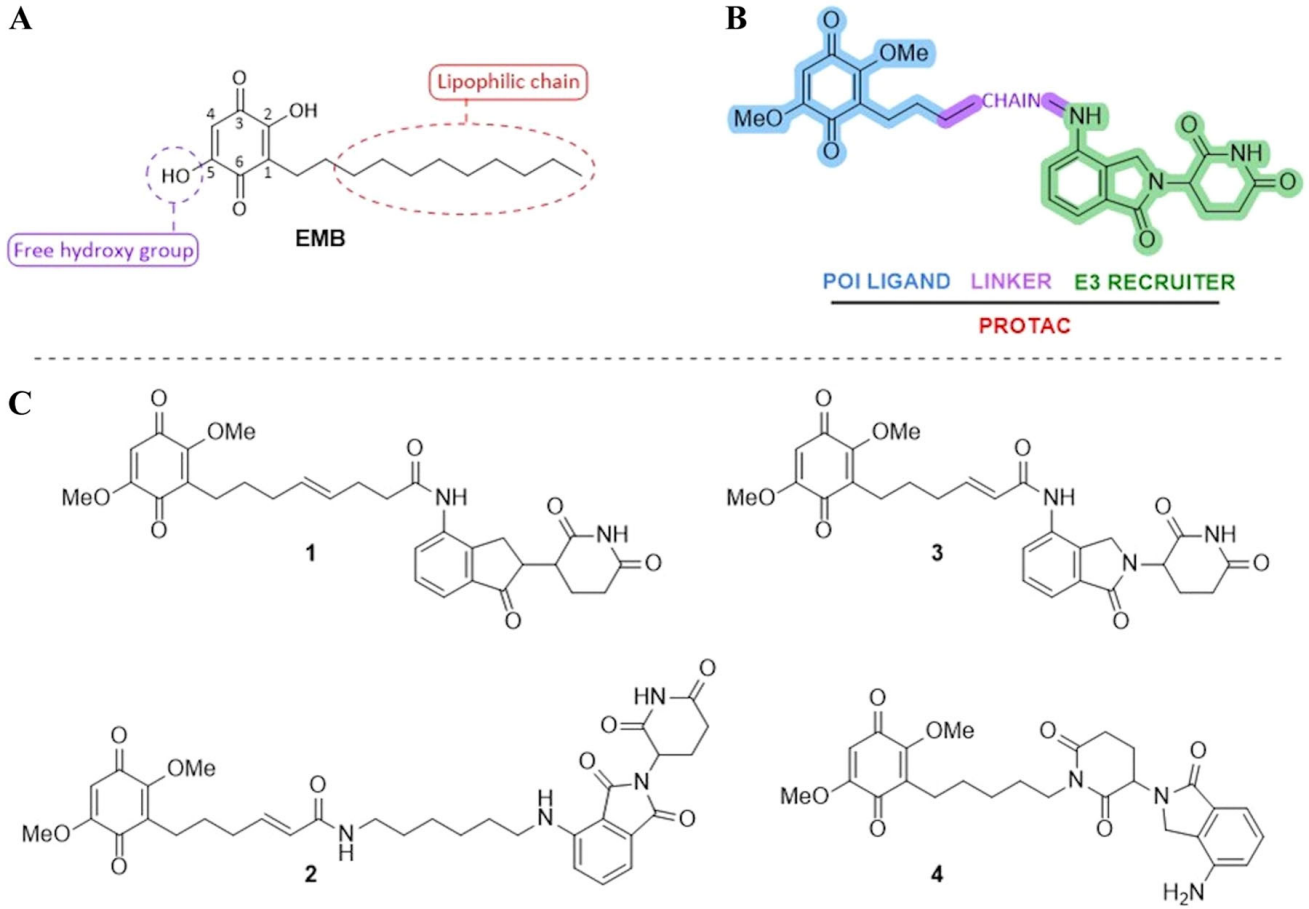
Conceptualisation of PROTAC compounds to enhance the degradation of neuroserpin. (A) Main areas of chemical intervention on the lead compound EMB (POI: protein of interest). (B) General PROTAC structure of the compounds conceived in this work. (C) Chemical structures of the four putative PROTACs to be synthesised.

We focused on the small molecule embelin (EMB) as a lead compound based on our previous studies, describing the interaction between EMB and NS (Saga et al. 2016), and reporting an analysis of the structure–activity relationship (SAR) for EMB binding to NS (Visentin et al. 2020). Different EMB regions can be used to build the PROTAC construct, i.e. the lipophilic lateral chain, the free hydroxy group in position 5, and position 4 (Figure 2A). We focused on the functionalization of the lipophilic lateral chain of EMB to introduce a proper functional group for linker conjugation and for the construction of the POI ligand‐linker‐E3 recruiter compounds (PROTAC, Figure 2B), selecting lenalidomide and thalidomide as cereblon recruiters for the proteasomal degradation machinery. Furthermore, we chose the dimethoxylated EMB‐like derivative for its easier handling, considering that no difference in activity was observed (Visentin et al. 2020). Cross metathesis, amide bond formation, and nucleophilic substitution were employed for domain‐conjugation in the synthesis of four PROTAC‐type degraders, i.e. **1**, **3**, **4** with lenalidomide and **2** with thalidomide as recruiters (Figure 2C).

**Scheme 1:**
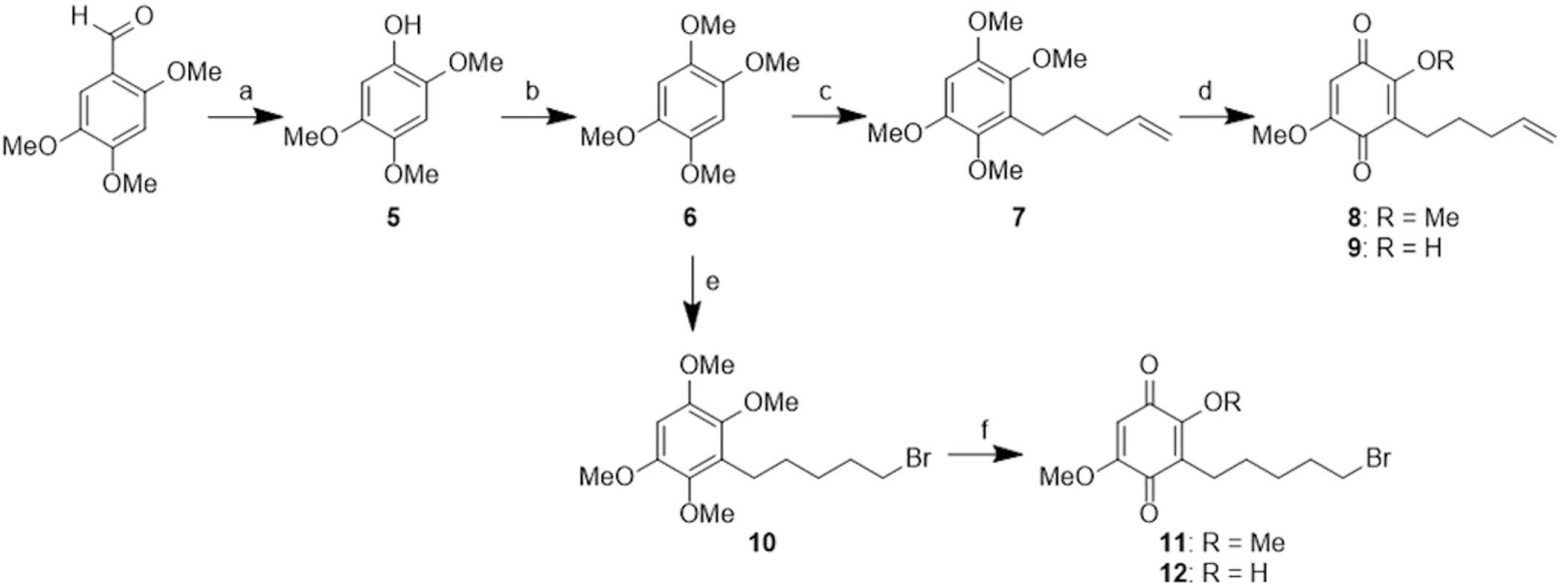
Synthesis of functionalized EMB‐like moieties **8** and **11**. **a** H_2_O_2_ 30%, H_2_SO_4_, MeOH, rt, 2 h, 84% yield; **b** MeI, Cs_2_CO_3_, ACN, 80°C, 5 h, 93% yield; **c** 1) *n*‐BuLi (1.6 M in THF), THF, 0°C to rt, 30 min, 2) 5‐bromo‐1‐pentene, 0°C to rt, 5 h, 58% yield; **d** CAN, ACN:H_2_O, ‐5°C to rt, 2 h, **8**: 32% yield, **9**: 41% yield; **e** 1) *n*‐BuLi (1.6 M in THF), THF, 0°C to rt, 30 min, 2) 1,5‐dibromopentane, 0°C to rt, 4 h, 60% yield; **f** CAN, ACN:H_2_O, rt, 2 h, **11**: 37% yield, **12**: 38% yield.

*Synthesis of POI ligand portions **8** and **11***. Firstly, commercially available 2,4,5‐trimethoxybenzaldehyde was converted into 2,4,5‐trimethoxyphenol **5** in high yield, using 30% hydrogen peroxide and sulfuric acid (Scheme 1, step a). The subsequent reaction with iodomethane (MeI) in basic condition gave **6** in almost quantitative yield (Scheme 1, step b). Tetramethoxybenzene **6** was used for the preparation of **8** and **11**, i.e. the key intermediates for the introduction of the linker portion.

According to our strategy, we aimed to functionalise the lateral chain of EMB by introducing either a terminal double bond or a bromine. Firstly, a directed *ortho*‐metalation (DoM) reaction was performed on tetramethoxybenzene **6** in presence of *n*‐BuLi as a strong base, and 5‐bromo‐1‐pentene as an electrophile, leading to 3‐(pent‐4‐en‐1‐yl)‐tetramethoxybenzene **7** (Scheme 1, step c) in a satisfactory yield, considering the unavoidable presence of a dialkylated by‐product. Then, intermediate **7** was oxidised using ceric ammonium nitrate (CAN), yielding both the di‐methoxy **8** and mono‐methoxy derivative **9** (Scheme 1, step d). Rather, the EMB lateral chain was functionalized with bromine with a similar DoM reaction, using 1,5‐dibromopentane as electrophile, achieving the desired Br‐tetramethoxybenzene **10** in a good yield (Scheme 1, step e), with a minor dialkylated by‐product. Intermediate **10** was oxidized with CAN, obtaining both the di‐methoxy **11** and mono‐methoxy derivative **12** (Scheme 1, step f).

**Scheme 2:**
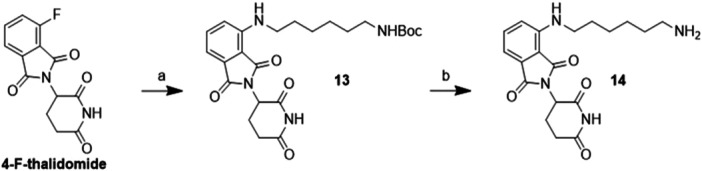
Synthesis of *N*‐thalidomide recruiter **14**: **a** DIPEA, DMF, 90°C, 6 h, 74% yield; **b** TFA, DCM, rt, 24 h, 95% yield.

*Synthesis of E3 recruiter portion **14***. Commercially available 4‐F‐thalidomide was subjected to a nucleophilic substitution with *N*‐Boc‐1,6‐hexanediamine (Scheme 2, step a), achieving the fluorescent *N*‐Boc‐thalidomide derivative **13** in a very good yield. Boc deprotection with trifluoroacetic acid (TFA) in dichloromethane (DCM) led to non‐protected intermediate **14** in almost quantitative yield (Scheme 2, step b). Rather, commercially available lenalidomide was used as such for the coupling with linker‐EMB moieties.

**Scheme 3:**
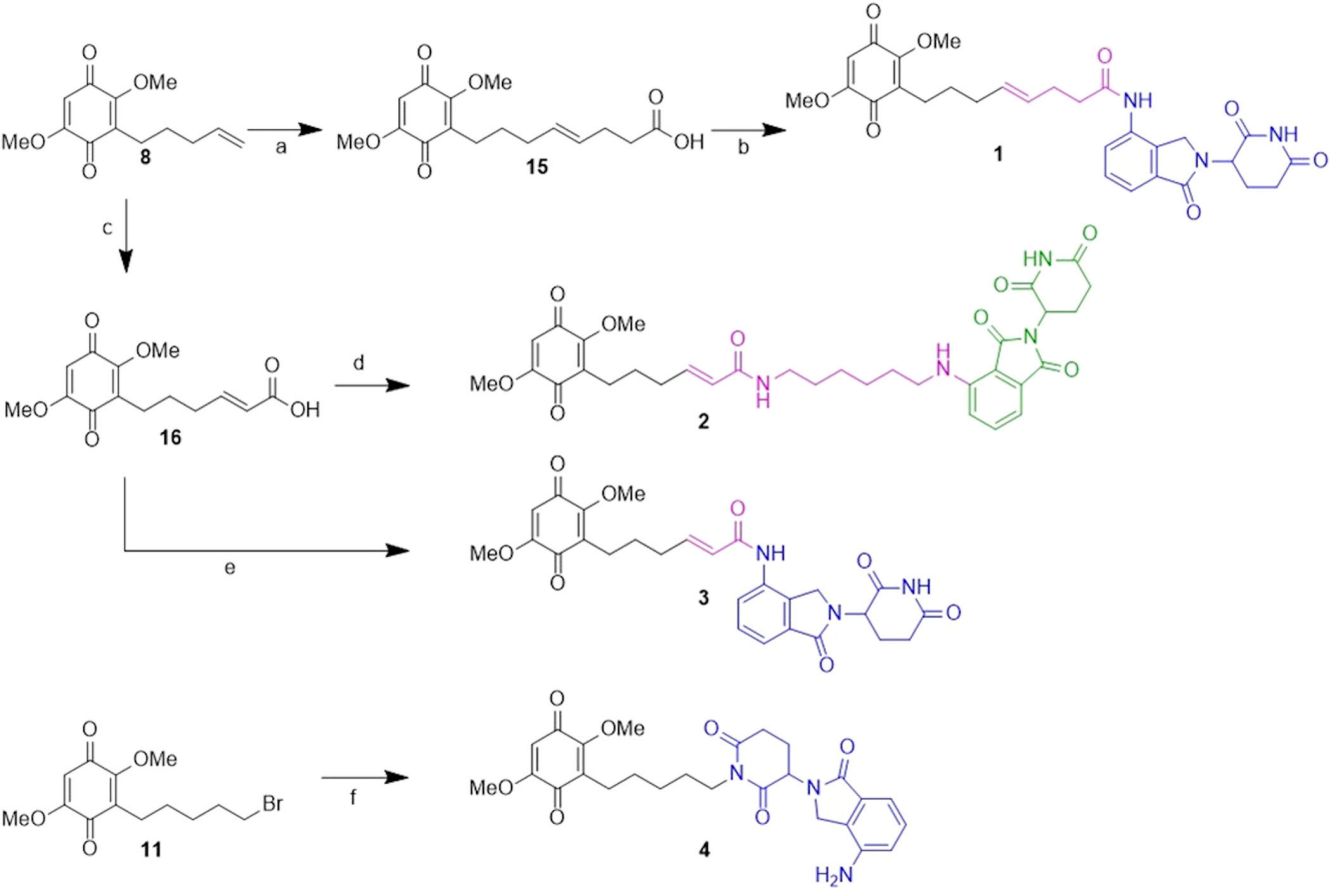
Synthesis of bivalent compounds **1*–*4**: **a** Pentenoic acid, Grubbs 2nd gen., DCM, 40°C, 6 h, 67% yield; **b** Lenalidomide, HATU, DIPEA, DMF, rt, 24 h, 15% yield; **c** Acrylic acid, Grubbs 2nd gen., DCM, 40°C, 6 h, 84% yield; **d 14**, HATU, DIPEA, THF, rt, 24 h, 66% yield; **e** Lenalidomide, HATU, DIPEA, DMF, rt, 24 h, 14% yield; **f** K_2_CO_3_, DMF, 80°C, 15 min, 42% yield.

*Synthesis of bivalent compounds **1–4***. A cross‐metathesis reaction was carried out between EMB‐related **8** and pentenoic acid, giving the COOH‐EMB intermediate **15** (Scheme 3, step a), then subjected to the amide condensation with lenalidomide (Scheme 3, step b). Putative PROTAC **1** was obtained, even if in a very low, unoptimized yield. Then, cross metathesis was performed between EMB‐related **8** and acrylic acid, obtaining the desired intermediate **16** (Scheme 3, step c). The latter was reacted both with *N*‐thalidomide derivative **14** (Scheme 3, step d) and lenalidomide (Scheme 3, step e) with classical condensation procedures, achieving putative PROTACs **2** and **3**, respectively. The reaction between **11** and lenalidomide was performed with potassium carbonate (Scheme 3, step f); after only 10 min, the formation of putative PROTAC **4** was observed, thanks to the rapid acidic proton deprotonation and attack on the bromine.

### 3.3 Testing of Putative PROTAC Compounds That Target NS in the Inducible NPC‐NS Cell Lines

**Figure 3:**
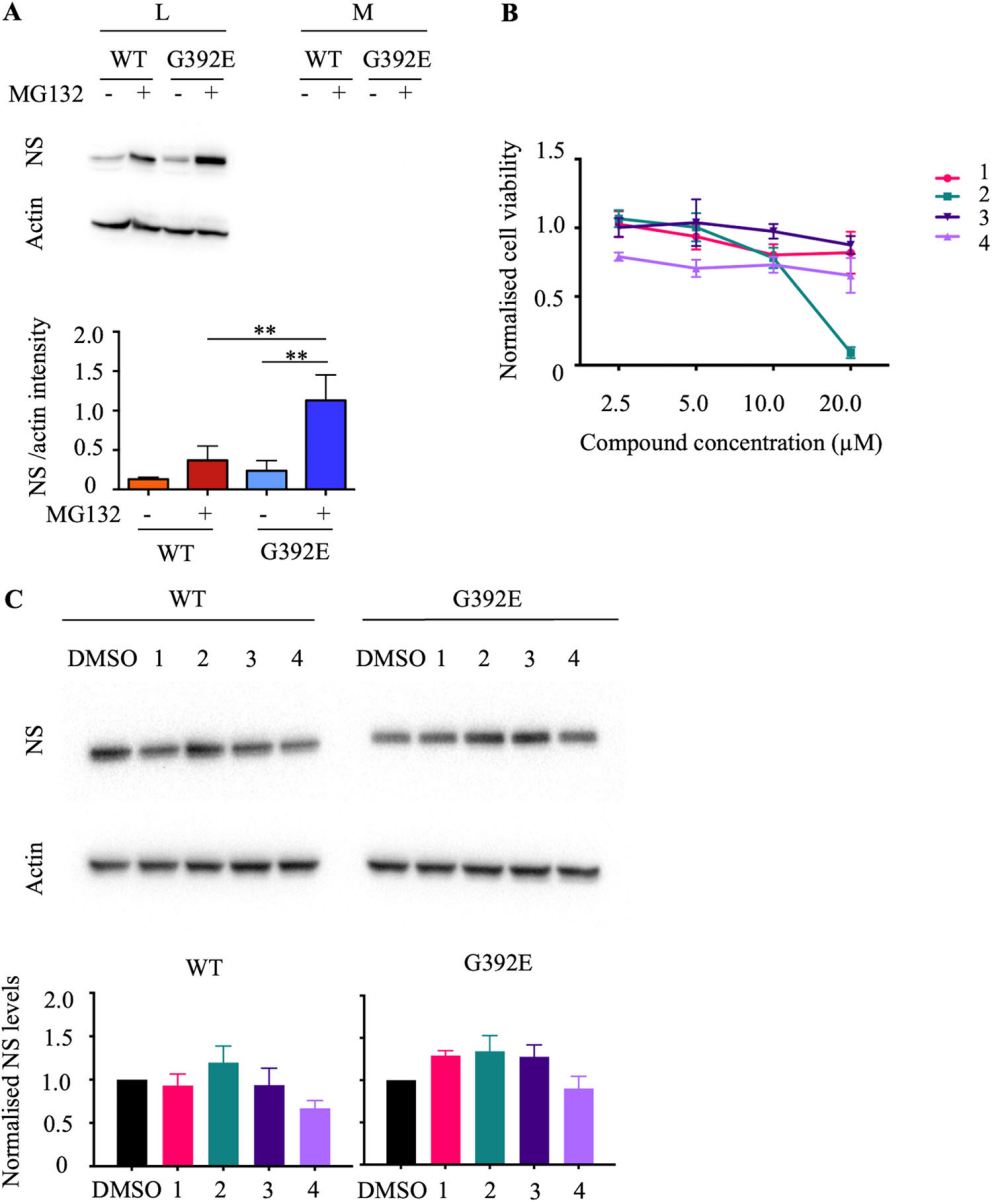
Testing of the putative PROTAC compounds in the inducible cellular model of FENIB. (A) Cell lysates and culture medium supernatants of NPCs expressing WT or G392E NS, induced with doxycycline 500 ng/mL for 7 h and treated or not with the proteasomal inhibitor MG132 2 µM for 6 h, were analysed by SDS‐PAGE and western blot using anti‐NS polyclonal and anti‐actin (loading control) antibodies. NS and actin levels were quantified by densitometry in three independent repeats and the results graphed as the average ± SD of NS signal normalised to actin. Statistical significance was assessed by ANOVA and Tukey's multiple comparisons test. (B) MTT assay to assess cell viability in the presence of PROTAC compounds 1 to 4. NPC G392E NS cells were treated with the four PROTAC compounds at 2.5, 5, 10 and 20 µM for 24 h, and subjected to MTT assay at the end of the incubation time. The abs at 560 nm for each condition was normalised to the control condition (DMSO only), and the data in the graph represent the average ± SD of three independent experiments. (C) NPC WT and G392E NS cells were treated with the four PROTAC compounds used at 10 µM, added 1 h after NS induction with doxycycline at 500 ng/mL, and incubated for 6 h. Cells were collected, lysed and analysed by SDS‐PAGE and western blot, and NS levels were quantified by densitometry. The graphs present the results from three independent experiments, presented as average + SD of NS signal normalised to actin and to the corresponding DMSO control.

To assess if variations in NS levels due to proteasomal degradation could be easily detected in our novel cellular model of FENIB, we induced NS expression in either NPC WT and G392E NS cells with 500 ng/mL doxycycline for 1 h, then for an additional 6 h either in the absence or in presence of the proteasomal inhibitor MG132 (2 μM). These conditions were selected after testing three different doxycycline concentrations (250, 500 and 1000 ng/mL) for 7 h, as a time and induction level appropriate for later testing the PROTAC molecules (data not shown). NS levels were quantified by SDS‐PAGE and western blot, followed by densitometry analysis of the NS signal in cell lysates and culture medium supernatants. The results presented in Figure 3A (western blot panels and densitometry graph) show the absence of secreted NS for both lines in this short‐induction condition, a nonsignificant increase in WT NS levels, and a strong increase in intracellular G392E NS levels that is statistically significant when compared to treated WT NS levels and to untreated G392E NS levels. These results confirm the detection of alterations in NS levels due to proteasomal manipulation in the conditions selected for PROTAC testing.

We then assessed the toxicity of PROTAC compounds at decreasing concentrations (20, 10, 5 and 2.5 µM) by adding them to NPC G392E NS cells 1 h after NS induction with doxycycline (500 ng/mL), and incubating them for 24 h. We reasoned that NPC G392E NS cells may be the most sensitive to PROTAC toxicity, due to their expression of a pathogenic mutant protein. NPC G392E NS cellular viability was quantified with the MTT (3‐(4,5‐dimethylthiazol‐2‐yl)‐ 2,5‐diphenyltetrazolium bromide) assay, in which the MTT salt is reduced to purple formazan crystals by mitochondrial dehydrogenases at a rate that correlates with the metabolic activity of the cells. As shown in Figure 3B, NPC G392E NS cells displayed high viability at all tested concentrations, except for PROTAC 2 at 20 µM. Therefore, we performed the following activity tests using putative PROTAC compounds at 10 µM concentration.

We next tested the four PROTAC compounds to assess their ability to enhance NS degradation by treating either NPC WT and G392E NS cells, using the conditions established in the proteasomal inhibition experiment with MG132 described above, with induction of NS expression with 500 ng/mL doxycycline, and addition of the PROTACs 1 h after NS induction, incubating for 6 h in the presence of doxycycline. Cells were lysed and analysed by denaturing PAGE and western blot, and NS levels were quantified by densitometry. As shown in Figure 3C, small differences in NS levels were detected with different treatments, but none of them were statistically significant against the negative control cells treated with DMSO (used to dilute the PROTAC molecules). These results indicate a lack of activity of the putative PROTAC compounds with regard to NS degradation by the proteasome.

## 4 Discussion

The neurodegenerative pathology FENIB is, currently, incurable. Although we have good knowledge of the molecular basis of this disease, effective therapeutic interventions are still lacking. Polymer formation by mutant NS is at the bases of neuronal toxicity, as shown by the presence of oxidative stress and mitochondrial alterations in mouse neurons overexpressing G392E NS (D'Acunto et al. 2022; Guadagno et al. 2017). We have described before that the small molecule EMB binds to monomeric WT NS forming a 1:1 complex, decreasing the formation of heat‐induced polymers and favouring the formation of small oligomers in vitro. In addition, EMB was also able to bind to preformed NS polymers and induce their breakdown to shorter oligomers (Saga et al. 2016). These encouraging results were tempered by the fact that EMB is poorly soluble in aqueous solutions and did not prevent G392E NS polymer formation when applied to a cellular model of FENIB based on transient expression of NS in COS‐7 cells (Visentin et al. 2020). Although a reduction in NS levels was observed, this was also true for WT NS and for a non‐related neuronal protein, neuroligin 3, and the EMB effects were reverted by incubating with the proteasomal inhibitor MG132, suggesting that EMB caused a nonspecific enhancement of proteasomal degradation. These results led us to explore the properties of EMB, by designing EMB analogues that could have improved its solubility and performance with regard to formation of NS polymers. None among our EMB analogues showed better properties than EMB, indicating that EMB optimally interacts with NS (Visentin et al. 2020).

Since EMB binds to NS, we explored the possibility of creating PROTAC compounds based on EMB to induce a proteasomal degradation of NS. We have shown earlier that a small portion of WT NS and a higher fraction of polymerogenic variants of NS that cause FENIB are degraded by the proteasome (Kroeger et al. 2009; Miranda, Römisch, and Lomas 2004), so we reasoned that an effective PROTAC compound could accelerate the proteasomal degradation of mutant NS after retrotranslocation to the cytosol. It is unlikely that ubiquitination and retrotranslocation of NS from the ER to the cytosol are the rate limiting steps for its proteasomal degradation. Moreover, these steps have been shown to depend on interactions with the ER‐associated E3 ligases Hrd1 and gp78 and with the VCP/p97 AAA ATPase (Ying et al. 2011). Still, it has been described in yeast that ERAD substrates are de‐ubiquitinated following retrotranslocation by VCP/p97, and are later re‐ubiquitinated by cytosolic E3 ligases for proteasomal delivery (Christianson, Jarosch, and Sommer 2023). This may be a step where a generalist cytosolic E3 ligase can engage with NS, and this interaction could be potentiated by a PROTAC molecule, therefore accelerating its proteasomal degradation and reducing the time of residence of unfolded NS in the cytosol. It has been recently reported that polymers of mutant Z alpha‐1 antitrypsin, a serpin that presents a mechanism of disease similar to FENIB, can be found within mitochondria, with increased amounts of mitochondrial accumulation upon proteasomal inhibition (Khodayari et al. 2021). Since it is highly unlikely that polymers can enter the mitochondria directly, a possible explanation is that unfolded alpha‐1 antitrypsin gets translocated from the cytosol and forms polymers in the mitochondrial matrix. Increasing the efficiency of proteasomal degradation would reduce the chances for this to occur, so it seems relevant to test PROTAC molecules against polymerogenic mutant serpins like alpha‐1 antitrypsin and NS. We thus synthesised four putative PROTAC compounds based on the dimethoxylated EMB as a ligand for NS, and thalidomide or lenalidomide as recruiters for the E3 ligase cereblon, which has been extensively used for PROTAC development (Ito 2024). The EMB portion was synthesised by modifying its lipophilic lateral chain, since it is not fundamental for its interaction with NS, while leaving the quinonic core intact. Linear linkers were employed as a first choice to ensure flexibility, and 5 to 12 carbon atom lengths were explored, while the use of thalidomide and lenalidomide as recruiters for cereblon offered different solubilities and easy functionalisation with amidation and nucleophilic substitution. Finally, we selected the cross‐metathesis reaction as a compatible conjugation strategy with the EMB quinone, which is rather sensitive to reactions involving reducing conditions. Unfortunately, the scarce solubility of these compounds has prevented us from performing the biophysical studies needed to demonstrate their binding to NS in vitro. For this reason, we decided to test our PROTAC compounds directly in a cellular system.

To test our putative PROTAC compounds, and to enable future efforts towards a pharmacological intervention against FENIB, we have created a novel model of FENIB with inducible expression of both WT NS and the G392E variant that causes severe FENIB. We selected NPCs as a proliferative but more physiological cellular model system for NS expression, and used the integrative, inducible bis‐cistronic ePB plasmid for NS expression. This plasmid allows the expression of a NS under the control of a doxycyclin‐inducible promoter, which also controls the expression of the antibiotic used for selection of cells with plasmid integration (puromycin). We show here that our novel cell model of FENIB recapitulated the handling of NS observed in our previous models of disease. Upon induction of NS expression, WT NS was readily secreted into the culture medium as a monomeric protein with the expected molecular weight. In contrast, G392E NS was found both in the intracellular fraction and culture medium as a ladder of polymeric chains of increasing size, and the intracellular fraction co‐localised with the ER retention peptide KDEL, as seen in COS‐7 and PC12 cells (Miranda et al. 2008). The novel cell model system showed no leaking expression of NS in the absence of doxycyclin, and we could dose NS expression by treating cells with different concentrations of doxycyclin. These properties allowed us to find the right conditions to obtain easily detectable NS levels within cells without reaching saturation, as shown by our ability to observe an increase in NS levels upon treatment with the proteasome inhibitor MG132. We selected 7 h as a short time of expression to avoid the accumulation of intracellular G392E NS polymers, to maximise the chances of detecting a change in NS levels upon treatment with the PROTAC compounds.

Once ascertained that the novel cell model of FENIB was appropriate for molecule testing, we treated G392E NS cells with the four PROTAC compounds to assess their toxicity, and found that only PROTAC **2** was toxic when applied at 20 µM for 24 h. This low toxicity allowed us to test all four PROTACs at 10 µM for 6 h, but we did not find any significant alterations in NS levels, either in WT or G392E NS expressing cells. This lack of effect may be due to several reasons. First, our PROTAC compounds may not penetrate into the cells, but the toxicity observed with PROTAC 2 suggests that at least this compound is cell‐permeable. Alternatively, once in the cytoplasm, our PROTACs may not be efficient in binding to cereblon and NS simultaneously; both recruiters used to construct our PROTAC compounds, EMB and thalidomide or lenalidomide, have been shown to be efficient in engaging NS and cereblon respectively, but a direct proof of simultaneous engagement is lacking. Furthermore, NS needs to refold at least partially once retro‐translocated to the cytosol to be bound by EMB, a process that has not been investigated so far. Future studies need to address these issues to continue the development of PROTAC therapeutics for FENIB.

In conclusion, we report here the creation and characterisation of a novel cell model system for FENIB with stable, inducible expression of either WT or G392E NS, which recapitulates the handling of WT and polymerogenic NS in combination with optimal control of NS expression. This cell system will be of great value in future efforts aimed to develop small molecule therapies for FENIB, aimed to increase NS degradation or to block polymer formation, as shown for a similar protein aggregation disease, alpha‐1 antitrypsin deficiency, for which a small molecule able to prevent alpha‐1 antitrypsin polymer formation has already been described (Lomas et al. 2021). We also describe the first attempt to produce and apply PROTAC compounds aimed to enhance the proteasomal degradation of mutated NS, opening the way to further work in this direction.

## Author Contributions

All authors have contributed to the conception and design of the study, or the acquisition of data, or the analysis and interpretation of data. All authors have contributed to the critical revision of the manuscript and have approved its final version. Alessandro Giustini, Alice Maiocchi, Ilaria Serangeli, Daniele Passarella and Elena Miranda have drafted the manuscript.

## Conflicts of Interest

The authors declare no conflicts of interest.

## Declaration of Generative AI and AI‐Assisted Technologies in the Writing Process

The authors declare that AI and AI‐assisted technologies have not been used in the writing of the manuscript.

## Supporting information

Supporting information.

## Data Availability

All the data generated in this study are reported in the figures that are part of this article. The original data are available from the corresponding authors at reasonable request.
