# Supplementary material for: An Inducible Neural Stem Progenitor Cell Model for Testing Therapeutic Interventions Against Neurodegeneration FENIB
Source: Drug Dev Res. 2025 Jan 3;86(1):e70041. doi: 10.1002/ddr.70041 (PMC11696822; doi:10.1002/ddr.70041)
Supplement: Supplementary file 1 — Supporting information. [file DDR-86-e70041-s001.docx]

**Supplementary methods**

**Synthesis of PROTAC compounds**

Oven-dried glassware was used to carry out chemical reactions, and dry solvents under a nitrogen atmosphere were employed. Solvents were purchased from Merck and used as such. Chemical reagents were purchased from Merck, Fluorochem and TCI and used without further purification. Reaction monitoring by thin layer chromatography (TLC) entailed Merck-precoated 60F_254_ plates, using UV light at 254 nm as a direct detection method, or by staining with a phosphomolybdic acid ethanolic solution. Purification of intermediates and final products was carried out by flash chromatography using high purity grade silica gel (Merck Grade, 60 Å pore size, 230–400 mesh particle size, Sigma-Aldrich, Milan, Italy) as a stationary phase. ^1^H-NMR and ^13^C-NMR spectra were recorded in CDCl_3_ on a Bruker AC 400 instrument. Chemical shifts (δ) for proton and carbon signals are quoted relatively to tetramethylsilane (TMS) as an internal standard, and expressed in parts per million (ppm). HRMS spectra were recorded using electrospray ionisation (ESI) on a FT-ICR APEXII (Bruker Daltonics, Bremen, Germany).

*Synthesis of 2,4,5-trimethoxyphenol* ***5***

30% H_2_O_2_ (15 mmol, 472 μL, 3.00 eq) and H_2_SO_4_ (0.10 mmol, 5 μL, 0.02 eq) were added dropwise to a solution of 2,4,5-trimethoxybenzaldehyde (1 g, 5.1 mmol, 1.00 eq) in MeOH (10 mL). The resulting mixture was stirred at rt for 3 hours, until the disappearance of the starting aldehyde, monitoring by TLC (eluent: *n*-Hex/EtOAc 6:4, developed in potassium permanganate). Then, water (10 mL) was added to the mixture and the solution was neutralized to pH 6-7. The aqueous phase was extracted with EtOAc (4 x 20 mL). The combined organic extracts were dried over anhydrous Na_2_SO_4_, filtered, and concentrated. The crude red oil was purified by flash chromatography (eluent: 6:4 *n*-Hex/EtOAc) to give pure 2,4,5-trimethoxyphenol **5** (788 mg, 4.3 mmol, 84% yield) as a white solid. ^1^H NMR (400 MHz, CDCl_3_) δ 6.60 (s, 1H), 6.57 (s, 1H), 5.27 (bs, 1H), 3.85 (s, 3H), 3.84 (s, 3H), 3.81 (s, 3H). Spectroscopic data are consistent with that reported in the literature (Lamblin et al., 2012).

*Synthesis of 1,2,4,5-tetramethoxybenzene* ***6***

Cs_2_CO_3_ (10.9 g, 33.6 mmol, 2 eq) and MeI (5.23 mL, 84.0 mmol, 5 eq) were added dropwise to a solution of 2,4,5-trimethoxyphenol **5** (3.1 g, 16.8 mmol, 1 eq) in ACN (56 mL). The resulting mixture was stirred at 80 °C for 6 hours and monitored by TLC (eluent: 6:4 *n*-Hex/EtOAc, developed in potassium permanganate). After reaction completion, water (20 mL) was added to the mixture. The aqueous phase was extracted with EtOAc (4 x 20 mL). The combined organic extracts were dried over anhydrous Na_2_SO_4_, filtered, and concentrated, affording a white crude solid. The crude was purified by flash chromatography (eluent: 6:4 *n*-Hex/EtOAc) achieving pure 1,2,4,5-tetramethoxybenzene **6** (3.1 g, 15.6 mmol, 93% yield) as a white solid. ^1^H NMR (400 MHz, CDCl_3_) δ 6.60 (s, 2H), 3.85 (s, 12H). Spectroscopic data are consistent with that reported in the literature (Bai et al., 2019).

*Synthesis of 3-(pent-4-en-1-yl)-1,2,4,5-tetramethoxybenzene* ***7***

1.6 M *n*-BuLi (1.90 mL, 3.0 mmol, 1.2 eq) was added to a solution of tetramethoxybenzene **6** (500 mg, 2.5 mmol, 1.0 eq) in dry THF (12.5 mL) at 0 °C under nitrogen atmosphere. The reaction mixture was stirred for 15 minutes at 0 °C, then for 15 minutes at rt. 5-Bromo-1-pentene (0.970 mL, 7.5 mmol, 3.0 eq) was added at 0 °C, the yellow reaction mixture was stirred at 0 °C for 30 minutes and for 4 hours at rt. The reaction was monitored by TLC (eluent: 9:1 *n*-Hex/EtOAc, developed in molybdic reagent). Then, water (10 mL) was added, and the aqueous layer was extracted with EtOAc (4 x 20 mL). The combined organic layers were dried with Na_2_SO_4_, filtered, and evaporated under reduced pressure, giving a pale-yellow crude oil. The crude was purified by flash column chromatography (eluent: 9:1 *n*-Hex/EtOAc) to give pure **7** (380 mg, 1.4 mmol, 58% yield) as a colorless oil. MS (ESI^+^): m/z 267.1745 [M + H]^+^; calculated MS for C_15_H_23_O_4_: 267.1596. ^1^H NMR (400 MHz, CDCl_3_) δ 6.41 (s, 1H), 5.81 (ddt, *J* = 16.9, 10.3, 6.6 Hz, 1H), 5.06-4.93 (m, 2H), 3.84 (s, 6H), 3.76 (s, 6H), 2.65-2.61 (m, 2H), 2.10 (q, *J* = 7.2 Hz, 2H), 1.61 (p, *J* = 7.6 Hz, 2H). ^13^C NMR (101 MHz, CDCl_3_) δ 149.0, 141.2, 138.9, 130.6, 114.4, 96.9, 60.9, 56.2, 34.1, 30.0, 24.3.

*Synthesis of 3-(5-bromopentyl)-1,2,4,5-tetramethoxybenzene* ***10***

1.6 M *n-*BuLi (1.6 mL, 3.0 mmol, 1.2 eq) was added to a solution of tetramethoxybenzene **6** (500 mg, 2.5 mmol, 1.0 eq) in dry THF (12.6 mL) at 0 °C under nitrogen atmosphere. The reaction mixture was stirred for 15 minutes at 0 °C, then for 15 minutes at rt. 1,5-Dibromopentane (1.0 mL, 7.5 mmol, 3.0 eq) was added at 0 °C, the yellow reaction mixture was stirred at 0 °C for 30 minutes and for 3 h at rt. The reaction was monitored by TLC (eluent: 9:1 *n*-Hex/EtOAc, developed in molybdic reagent). Then, water (10 mL) was added, and the aqueous layer was extracted with EtOAc (4 x 20 mL). The combined organic layers were dried with Na_2_SO_4_, filtered, and evaporated under reduced pressure, giving a pale-yellow crude oil. The crude was purified by flash column chromatography (eluent: 9:1 *n*-Hex/EtOAc) to give pure **10** (519 mg, 1.5 mmol, 60% yield) as a colorless oil. MS (ESI^+^): m/z 347.0905 [M + H]^+^. Calculated MS for C_15_H_24_BrO_4_: 347.0858. ^1^H NMR (400 MHz, CDCl_3_) δ 6.41 (s, 1H), 3.84 (s, 6H), 3.76 (s, 6H), 3.40 (t, *J* = 6.8 Hz, 2H), 2.64-2.61 (m, 2H), 1.93-1.86 (m, 2H), 1.60-1.48 (m, 4H). ^13^C NMR (101 MHz, CDCl_3_) δ 149.3, 141.4, 130.9, 97.2, 61.4, 56.7, 34.3, 33.1, 30.1, 28.7, 24.7.

*Synthesis of 2,5-dimethoxy-3-(pent-4-en-1-yl)cyclohexa-2,5-diene-1,4-dione* ***8***

CAN (1.36 g, 2.5 mmol, 2 eq) was dissolved in 1:1 ACN:H_2_O (12.4 mL) and added dropwise to a solution of **7** (330 mg, 1.2 mmol, 1 eq) in ACN (12.4 mL) at -10 °C. The reaction was warmed under stirring to rt for 3 hours and monitored by TLC (eluent: *n*-Hex/EtOAc 7:3). After reaction completion, the aqueous phase was extracted with EtOAc (4 x 10 mL). The organic layer was washed with water (20 mL), brine (20 mL), dried over Na_2_SO_4_ and concentrated under vacuum. The orange crude oil was purified by flash chromatography (eluent: 8:2 to 7:3 *n*-Hex/EtOAc) to give the corresponding pure mono-methoxylated **9** (113 mg, 0.508 mmol, 41% yield) and di-methoxylated **8** (95 mg, 0.402 mmol, 32% yield) benzoquinones, as orange solid and orange oil respectively. **8**: MS (ESI^+^): m/z 237.1342 [M + H^+^]; calculated MS for C_13_H_17_O_4_: 237.1126. ^1^H NMR (400 MHz, CDCl_3_) δ 5.86-5.76 (m, 1H), 5.72 (s, 1H), 5.04-4.94 (m, 2H), 4.05 (s, 3H), 3.80 (s, 3H), 2.46-2.42 (m, 2H), 2.10-2.04 (m, 2H), 1.54-1.46 (m,2H). ^13^C NMR (101 MHz, CDCl_3_) δ 183.5, 182.4, 158.8, 155.9, 138.3, 130.2, 114.8, 105.4, 61.4, 56.4, 33.6, 27.8, 22.6. **9**: MS (ESI^+^): m/z 223.0987 [M + H]^+^; calculated MS for C_12_H_15_O_4_: 223.0970. ^1^H NMR (400 MHz, CDCl_3_) δ 5.84 (s, 1H), 5.86-5.76 (m, 1H), 5.04-4.93 (m, 2H), 3.85 (s, 3H), 2.47-2.44 (m, 2H), 2.11- 2.04 (m, 2H), 1.56 (p, *J* = 7.6 Hz, 2H). ^13^C NMR (101 MHz, CDCl_3_) δ 182.8, 181.6, 161.2, 151.7, 138.7, 118.9, 114.5, 102.3, 56.6, 33.5, 27.1, 22.3.

*Synthesis of 3-(5-bromopentyl)-2,5-dimethoxycyclohexa-2,5-diene-1,4-dione* ***11***

CAN (434 mg, 0.792 mmol, 2.5 eq) in 1:1 ACN:H_2_O (3.2 mL) was added dropwise to a solution of **10** (110 mg, 0.317 mmol, 1.0 eq) in ACN (3.2 mL) at -10 °C. The reaction was warmed under stirring to rt for 3 hours and monitored by TLC (eluent: 7:3 *n*-Hex/EtOAc). After reaction completion, the aqueous phase was extracted with EtOAc (4 x 10 mL). The organic layer was washed with water (20 mL), brine (20 mL), dried over Na_2_SO_4_ and concentrated under vacuum. The orange crude oil was purified by flash chromatography (eluent: 8:2 to 7:3 *n*-Hex/EtOAc) to give the corresponding pure mono-methoxylated **12** (36 mg, 0.119 mmol, 38% yield) and di-methoxylated **11** (37 mg, 0.117 mmol, 37% yield) benzoquinones, as orange solid and orange oil respectively. **11:** MS (ESI^+^): m/z 317.0118 [M + H]^+^. Calculated MS for C_13_H_18_BrO_4_: 317.0388. ^1^H NMR (400 MHz, CDCl_3_) δ 5.72 (s, 1H), 4.05 (s, 6H), 3.79 (s, 6H), 3.39 (t, *J* = 6.8, 1.4 Hz, 2H), 2.46-2.41 (m, 2H), 1.90-1.83 (m, 2H), 1.47-1.40 (m, 4H). ^13^C NMR (101 MHz, CDCl_3_) δ 183.5, 182.4, 158.7, 156.0, 129.8, 105.5, 61.4, 56.4, 33.8, 32.4, 28.0, 27.6, 22.8. **12:** MS (ESI^+^): m/z 303.0115 [M + H]^+^. Calculated MS for C_12_H_16_BrO_4_: 303.0232. ^1^H NMR (400MHz, CDCl_3_) δ 5.85 (s, 1H), 3.86 (s, 3H), 3.40 (t, *J* = 6.8 Hz, 2H), 2.46 (t, *J* = 7.2 Hz, 2H), 1.90-1.87 (m, 2H), 1.53-1.45 (m, 4H). ^13^C NMR (101MHz, CDCl_3_) δ 182.8, 181.7, 161.6, 151.3, 118.1, 102.4, 56.9, 33.8, 32.5, 27.9, 27.1, 22.4.

*Synthesis of tert-butyl (6-((2-(2,6-dioxopiperidin-3-yl)-1,3-dioxoisoindolin-4-yl)amino)hexyl)carbamate* ***13***

*N*-Boc-1,6-hexanediamine (70 mg, 0.44 mmol, 1.1 eq) was added to a stirred solution of 4-F-thalidomide (110 mg, 0.40 mmol, 1.0 eq) in dry DMF (5 mL) and DIPEA (0.200 mL, 1.2 mmol, 3.0 eq), under nitrogen atmosphere. The reaction mixture was stirred at 90 °C for 5 hours and monitored by TLC (eluent: 9:1 DCM/EtOAc). After reaction completion, the mixture was cooled to rt, poured into water (10 mL) and extracted with EtOAc (4 x 20 mL). The combined organic layer was washed with brine (20 mL), dried over anhydrous Na_2_SO_4_ and concentrated under reduced pressure. The crude fluorescent yellow oil was purified by flash chromatography (eluent: 9:1 DCM/EtOAc) to give pure **13** (140 mg, 0.296 mmol, 74% yield) as a fluorescent yellow oil. MS (ESI^+^): m/z 473.2562 [M + H]^+^. Calculated MS for C_24_H_33_N_4_O_6_: 473.2400. ^1^H NMR (400 MHz, CDCl_3_) δ 8.44 (s, 1H), 7.47 (m, 1H), 7.06 (d, *J* = 7.1 Hz, 1H), 6.86 (d, *J* = 8.5 Hz, 1H), 4.92-4.88 (m, 1H), 3.24 (t, *J* = 7.1 Hz, 2H), 3.09-3.12 (m, 2H), 2.83-2.89 (m, 1H), 2.67-2.77 (m, 2H), 2.09-2.12 (m, 1H), 1.64 (p, *J* = 7.1 Hz, 2H), 1.42-1.50 (m, 2H), 1.38 (s, 9H), 1.32-1.37 (m, 2H). ^13^C NMR (101 MHz, CDCl_3_) δ 171.0, 169.7, 168.4, 167.8, 156.2, 147.1, 136.3, 132.7, 116.8, 111.6, 110.0, 49.0, 42.7, 31.6, 30.2, 29.9, 29.3, 28.6, 26.8, 26.6, 23.0.

*Synthesis of 4-((6-aminohexyl)amino)-2-(2,6-dioxopiperidin-3-yl)isoindoline-1,3-dione* ***14***

A solution of **13** (140 mg, 0.305 mmol, 1 eq) and TFA (1.4 mL, 18.3 mmol, 60 eq) in dry DCM (1 mL) was stirred at rt for 24 hours. The solvent and TFA were evaporated under reduced pressure to give the corresponding deprotected intermediate **14** (108 mg, 0.291 mmol, 95% yield). MS (ESI^+^): m/z 373.1855 [M + H^+^]; calculated MS for C_19_H_24_N_4_O_4_: 373.1876. ^1^H NMR (400 MHz, MeOD) δ 7.53 (m, 1H), 7.03-7.00 (m, 2H), 5.07 (dd, *J* = 12.4, 5.4 Hz, 1H), 3.34-3.30 (m, 2H), 2.98-2.93 (m, 2H), 2.89-2.84 (m, 1H), 2.80-2.71 (m, 2H), 2.16-2.11 (m, 1H), 1.74-1.65 (m, 4H), 1.49-1.45 (m, 4H). ^13^C NMR (101 MHz, MeOD) δ 174.8, 171.8, 170.9, 169.5, 148.3, 137.4, 134.0, 118.2, 112.0, 111.1, 50.4, 43.4, 40.8, 32.4, 30.2, 28.6, 27.6, 27.3, 23.9.

*Synthesis of (E)-8-(2,5-dimethoxy-3,6-dioxocyclohexa-1,4-dien-1-yl)oct-4-enoic acid* ***15***

Grubbs 2^nd^ generation catalyst (10 mg, 0.012 mmol, 0.05 eq) was added to a stirred solution of pentenoic acid (0.054 mL, 0.482 mmol, 2.00 eq) in dry DCM (0.60 mL) under nitrogen atmosphere. A solution of the alkene **8** (57 mg, 0.241 mmol, 1.00 eq) in dry DCM (0.60 mL) was then added slowly dropwise. The reaction mixture was heated at reflux for 6 hours and monitored by TLC (eluent: 6:4 *n*-Hex/EtOAc, developed in potassium permanganate). After completion, the reaction mixture was concentrated and purified by flash column chromatography (eluent: 6:4 *n*-Hex/EtOAc + 0.05% AcOH) to give pure acid **15** (50 mg, 0.162 mmol, 67% yield) as a yellow oil. MS (ESI^+^): m/z 309.1510 [M + H]^+^; calculated MS for C_16_H_21_O_6_: 309.1338. ^1^H NMR (400 MHz, CDCl_3_) δ 5.72 (s, 1H), 5.50-5.40 (m, 2H), 4.04 (s, 3H), 3.79 (s, 3H), 2.44-2.37 (m, 4H), 2.33-2.29 (m, 2H), 2.01-1.97 (m, 2H), 1.44 (p, *J* = 7.6 Hz, 2H). ^13^C NMR (101 MHz, CDCl_3_) δ 183.7, 182.6, 177.8, 158.9, 156.1, 131.4, 130.4, 128.4, 105.6, 61.5, 56.5, 34.0, 32.5, 28.4, 27.8, 22.7.

*Synthesis of (E)-6-(2,5-dimethoxy-3,6-dioxocyclohexa-1,4-dien-1-yl)hex-2-enoic acid* ***16***

Grubbs 2^nd^ generation catalyst (5.4 mg, 0.006 mmol, 0.05 eq) was added to a stirred solution of acrylic acid (0.018 mL, 0.254 mmol, 2.00 eq) in dry DCM (0.62 mL) under nitrogen atmosphere. A solution of alkene **8** (30 mg, 0.127 mmol, 1.00 eq) in dry DCM (0.620 mL) was then added slowly dropwise. The reaction mixture was heated at reflux for 6 hours and monitored by TLC (eluent: *n*-Hex/EtOAc 6:4, developed in potassium permanganate). After completion, the reaction mixture was concentrated and purified by flash column chromatography (eluent: 6:4 *n*-Hex/EtOAc + 0.05% AcOH) to give pure acid **16** (30 mg, 0.107 mmol, 84% yield) as a yellow oil. MS (ESI^+^): m/z 281.1273 [M + H]^+^; calculated MS for C_14_H_17_O_6_: 281.1025. ^1^H NMR (400 MHz, CDCl_3_) δ 7.07 (dt, *J* = 15.6, 6.8 Hz, 1H), 5.85 (d, *J* = 15.6 Hz, 1H), 5.74 (s, 1H), 4.08 (s, 3H), 3.80 (s, 3H), 2.49-2.46 (m, 2H), 2.28-2.22 (m, 2H), 1.60 (p, *J* = 7.6 Hz, 2H). ^13^C NMR (101 MHz, CDCl_3_) δ 183.8, 182.6, 172.2, 159.2, 156.5, 151.8, 129.4, 121.4, 105.9, 61.9, 56.9, 32.4, 27.0, 22.9.

*(E)-8-(2,5-dimethoxy-3,6-dioxocyclohexa-1,4-dien-1-yl)-N-(2-(2,6-dioxopiperidin-3-yl)-1-oxoisoindolin-4-yl)oct-4-enamide* ***1***

Lenalidomide (28 mg, 0.108 mmol, 1.0 eq) and **15** (50 mg, 0.162 mmol, 1.5 eq) were dissolved in dry DMF (1 mL) under nitrogen atmosphere. HATU (41 mg, 0.108 mmol, 1.0 eq) and DIPEA (0.056 mL, 0.324 mmol, 3.0 eq) were then added, the mixture was stirred overnight at rt and monitored by TLC (eluent: 95:5 DCM/MeOH). The mixture was diluted with water (5 mL) and the aqueous phase was extracted with EtOAc (4 x 10 mL). The organic layer was washed with brine (20 mL), dried over Na_2_SO_4_ and concentrated under vacuum. The crude oil was purified by flash chromatography (eluent: 95:5 DCM/MeOH) to give pure **1** (9 mg, 0.016 mmol, 15% yield). MS (ESI^+^): m/z 550.2330 [M + H] ^+^; calculated MS for C_29_H_31_N_3_O_8_: 550.2189. ^1^H NMR (400 MHz, CDCl_3_) δ 8.26 (s, 1H), 7.71 (d, *J* = 7.5 Hz, 1H), 7.58 – 7.55 (m, 2H), 7.45 (t, *J* = 7.5 Hz, 1H), 5.72 (s, 1H), 5.54 - 5.48 (m, 2H), 5.18 (dd, *J* = 13.2, 5.2 Hz, 1H), 4.40 - 4.30 (m, 2H), 4.05 (s, 3H), 3.78 (s, 3H), 2.89 (d, *J* = 17.2 Hz, 1H), 2.84 - 2.75 (m, 1H), 2.49 - 2.38 (m, 6H), 2.37 - 2.28 (m, 1H), 2.24 – 2.17 (m, 1H), 2.03 – 1.99 (m, 2H), 1.51-1.43 (m, 2H). ^13^C NMR (101 MHz, CDCl_3_) δ 183.2, 171.5, 159.4, 156.7, 135.1, 133.3, 131.9, 129.3, 128.7, 126.4, 121.6, 105.7, 56.6, 52.0, 46.8, 37.1, 32.4, 31.7, 30.2, 28.7, 28.2, 23.6, 22.6 (detected signals).

*(E)-6-(2,5-dimethoxy-3,6-dioxocyclohexa-1,4-dien-1-yl)-N-(6-((2-(2,6-dioxopiperidin-3-yl)-1,3-dioxoisoindolin-4-yl)amino)hexyl)hex-2-enamide* ***2***

*N*-thalidomide derivative **14** (49 mg, 0.137 mmol, 1.0 eq) and **16** (50 mg, 0.178 mmol, 1.3 eq) were dissolved in dry THF (1.4 mL), under nitrogen atmosphere. Then, HATU (52 mg, 0.137 mmol, 1.0 eq) and DIPEA (0.050 mL, 0.274 mmol, 2.0 eq) were added, the mixture was stirred overnight at rt and monitored by TLC (eluent: 95:5 DCM/MeOH). The mixture was diluted with water (5 mL) and the aqueous phase was extracted with EtOAc (4 x 20 mL). The organic layer was washed with brine (20 mL), dried over Na_2_SO_4_ and concentrated under vacuum. The yellow crude solid was purified by flash chromatography (eluent: DCM/MeOH 95:5) to give pure **2** (58 mg, 0.091 mmol, 66% yield) as a yellow solid. MS (ESI^+^): m/z 635.2503 [M + H]^+^; calculated MS for C_33_H_39_N_4_O_9_: 635.2717. ^1^H NMR (400 MHz, CDCl_3_) δ 7.50 – 7.46 (m, 1H), 7.07 (d, *J* = 7.1 Hz, 1H), 6.87 (d, *J* = 8.5 Hz, 1H), 6.85 - 6.78 (m, 1H), 5.81 (dt, *J* = 15.0, 1.6 Hz, 1H), 5.74 (s, 1H), 4.95-4.90 (m, 1H), 4.07 (s, 3H), 3.81 (s, 3H), 3.33-3.24 (m, 4H), 2.87 - 2.86 (m, 1H), 2.79 - 2.73 (m, 2H), 2.47 – 2.44 (m, 2H), 2.25 - 2.11 (m, 3H), 1.66 (p, *J* = 6.9 Hz, 2H), 1.56 (h, *J* = 7.8 Hz, 4H), 1.47 - 1.36 (m, 4H). ^13^C NMR (101 MHz, CDCl_3_) δ 183.4, 182.4, 171.4, 169.5, 168.7, 167.6, 166.1, 158.8, 156.1, 147.0, 143.7, 136.1, 132.5, 129.4, 123.9, 116.7, 111.4, 109.9, 105.5, 61.4, 56.5, 48.9, 42.5, 39.4, 31.7, 31.4, 31.3, 29.5, 29.1, 26.9, 26.6, 22.8, 22.6.

*(E)-6-(2,5-dimethoxy-3,6-dioxocyclohexa-1,4-dien-1-yl)-N-(2-(2,6-dioxopiperidin-3-yl)-1-oxoisoindolin-4-yl)hex-2-enamide* ***3***

A mixture of lenalidomide (18 mg, 0.071 mmol, 1.0 eq) and **16** (20 mg, 0.071 mmol, 1.5 eq) was dissolved in 1:1 dry DCM/DMF (0.60 mL), under nitrogen atmosphere. EDCI (11 mg, 0.071 mmol, 1.0 eq) and HOBt (10 mg, 0.071 mmol, 1.0 eq) were then added, the mixture was stirred overnight at rt and monitored by TLC (eluent: 95:5 DCM/MeOH). The mixture was diluted with water (5 mL) and the aqueous phase was extracted with EtOAc (4 x 10 mL). The organic layer was washed with brine (10 mL), dried over Na_2_SO_4_ and concentrated under vacuum. The crude oil was purified by flash chromatography (eluent: 95:5 DCM/MeOH) to give pure **3** (5 mg, 0.01 mmol, 14% yield). MS (ESI^+^): m/z 544.1623 [M + Na]^+^; calculated MS for C_27_H_27_N_3_O_8_: 544.1696. ^1^H NMR (400 MHz, MeOD) δ 7.86 (d, *J* = 7.5 Hz, 1H), 7.75 (d, *J* = 7.5 Hz, 1H), 7.62 (t, *J* = 7.5 Hz, 1H), 7.05 (dt, *J* = 15.0, 6.7 Hz, 1H), 6.26 (d, *J* = 15.0 Hz, 1H), 5.95 (s, 1H), 5.26 (dd, *J* = 13.2, 5.3 Hz, 2H), 4.64 – 4.55 (m, 2H), 4.16 (s, 3H), 3.89 (s, 3H), 3.05 – 2.95 (m, 1H), 2.90 – 2.86 (m, 1H), 2.67 – 2.63 (m, 1H), 2.59 (t, *J* = 7.5 Hz, 2H), 2.41 – 2.36 (m, 2H), 2.30 – 2.27 (m, 1H), 1.78 – 1.71 (m, 2H). ^13^C NMR (101 MHz, MeOD) 174.6, 172.1, 171.1, 147.6, 136.3, 136.0, 134.0, 130.1, 127.5, 124.2, 121.5, 106.5, 61.6, 57.1, 53.7, 48.3, 32.9, 32.4, 28.2, 24.1, 23.6 (detected signals).

*3-(4-amino-1-oxoisoindolin-2-yl)-1-(5-(2,5-dimethoxy-3,6-dioxocyclohexa-1,4-dien-1-yl)pentyl)piperidine-2,6-dione* ***4***

Lenalidomide was added dropwise to a solution of **10** (30 mg, 0.095 mmol, 1 eq) and K_2_CO_3_ (65 mg, 0.475 mmol, 5 eq) in dry DMF (0.400 mL). Then, the reaction mixture was warmed to 80 °C and stirred for 15 minutes, monitoring by TLC (eluent: 92:8 DCM/MeOH, developed in ninhydrin). The mixture was concentrated under reduced pressure and purified by flash column chromatography (eluent: 98:2 DCM/MeOH) to afford pure **4** (20 mg, 0.040 mmol, 42% yield). MS (ESI^+^): m/z 496.2113 [M + H]^+^; calculated MS for C_26_H_30_N_3_O_7_: 496.2083. ^1^H NMR (400 MHz, CDCl_3_) δ 7.28-7.22 (m, 2H), 6.83 (dd, *J* = 7.1, 1.7 Hz, 1H), 5.69 (s, 1H), 5.16 (dd, *J* = 13.4, 5.2 Hz, 1H), 4.30-4.14 (m, 2H), 4.01 (s, 3H), 3.79-3.70 (m, 2H), 3.74 (s, 3H), 2.90-2.89 (m, 1H), 2.82-2.76 (m, 1H), 2.39 (t, *J* = 7.4 Hz, 2H), 2.23 (qd, *J* = 13.1, 4.8 Hz, 1H), 2.14-2.09 (m, 1H), 1.54-1.46 (m, 2H), 1.42-1.34 (m, 2H), 1.28-1.20 (m, 2H). ^13^C NMR (101 MHz, CDCl_3_) δ 183.6, 182.5, 171.2, 170.1, 169.9, 158.8, 156.1, 141.4, 132.4, 130.1, 129.5, 126.5, 118.2, 114.3, 105.5, 61.5, 56.5, 52.5, 45.1, 40.4, 32.2, 28.2, 27.7, 26.7, 22.9, 22.8.

**References**

Bai, Y., He, X., Bai, Y., Sun, Y., Zhao, Z., Chen, X., Li, B., Xie, J., Li, Y., Jia, P., Meng, X., Zhao, Y., Ding, Y., Xiao, C., Wang, S., Yu, J., Liao, S., Zhang, Y., Zhu, Z., … Zheng, X. (2019). Polygala tenuifolia-Acori tatarinowii herbal pair as an inspiration for substituted cinnamic α-asaronol esters: Design, synthesis, anticonvulsant activity, and inhibition of lactate dehydrogenase study. *European Journal of Medicinal Chemistry*, *183*, 111650. https://doi.org/10.1016/j.ejmech.2019.111650

Lamblin, M., Sallustrau, A., Commandeur, C., Cresteil, T., Felpin, F.-X., & Dessolin, J. (2012). Synthesis and biological evaluation of hydrophilic embelin derivatives. *Tetrahedron*, *68*(24), 4655–4663. https://doi.org/10.1016/j.tet.2012.04.024
